# 990 Flap Coverage for Fournier’s Gangrene

**DOI:** 10.1093/jbcr/iraf019.521

**Published:** 2025-04-01

**Authors:** Kristine Laing, Alwaleed Alammar, David Wallace, Alan Rogers

**Affiliations:** Ross Tilley Burn Centre, Sunnybrook Health Sciences Centre; Ross Tilley Burn Centre, Sunnybrook Health Sciences Centre; Ross Tilley Burn Centre, Sunnybrook Health Sciences Centre; Ross Tilley Burn Centre, Sunnybrook Health Sciences Centre

## Abstract

**Introduction:**

Fournier’s gangrene (FG) is a life-threatening necrotizing soft tissue infection (NSTI) affecting the perineum and genital region, necessitating urgent debridement and ultimately coverage, often in the burn center context. Skin grafts are often used for reconstruction but may result in suboptimal outcomes. Flap-based reconstructions have shown potential for better functional and aesthetic results, but no systematic review has been undertaken to date.

**Methods:**

This systematic review was conducted following the PRISMA protocol. A comprehensive search of EMBASE, MEDLINE, Cochrane, Google Scholar, and CINAHL databases was performed. The search terms included “Necrotizing soft tissue infections,” “Gas gangrene,” “Fournier’s gangrene,” “Flap,” and “Graft.” Covidence software facilitated study selection and reference tracking. The inclusion criteria targeted studies on human subjects involving flap reconstruction for NSTI defects of the perineum, while exclusion criteria eliminated studies referred to graft reconstruction, trauma and oncology defects. All study designs were included. A charting form was developed to categorize studies by variables such as study type, flap type, pathology, and defect location.

**Results:**

After removal of duplicates, the title and abstract of 3760 studies were assessed and 522 studies underwent full-text review. Of the 99 studies included in the qualitative synthesis and data extraction, 31% (n=31) were retrospective and 56% (n=55) were case reports/series.

Of the 395 cases described, 501 flaps used 59 distinct techniques, with the anterolateral thigh, medial thigh, and pudendal thigh flaps the most common. Only 2 free flaps were performed. Gracilis was the most frequent locoregional option; others included the anterolateral thigh, medial circumflex femoral perforator, internal pudendal artery perforator, and groin flaps. Complication rates were relatively low, with only 10 partial and 6 total flap losses reported. The majority of studies were published in plastic surgery journals, followed by urology journals; only one appeared in a burn journal. Notably, 58 studies (59%) were published within the last decade.

**Conclusions:**

After debridement, the aim of FG management is to obtain durable coverage, with a view to minimize disability and optimize appearance. Although skin grafting often fulfils this role, techniques higher on the reconstructive ladder, including local, regional and free flaps, are sometimes required. This systematic review demonstrates that flap-based reconstruction is a reliable method for managing Fournier’s gangrene defects.

**Applicability of Research to Practice:**

NSTI is commonly and effectively managed in the burn center setting. This systematic review highlights the important contribution of the plastic surgeon as part of multidisciplinary teams managing complex defects resulting from Fournier’s Gangrene.

**Funding for the Study:**

N/A

